# Complexation of the Mycotoxin Cyclopiazonic Acid with Lanthanides Yields Luminescent Products

**DOI:** 10.3390/toxins10070285

**Published:** 2018-07-10

**Authors:** Chris M. Maragos

**Affiliations:** Mycotoxin Prevention and Applied Microbiology Research Unit, Agricultural Research Service, U.S. Department of Agriculture, Peoria, IL 61604, USA; chris.maragos@ars.usda.gov; Tel.: +1-309-681-6266

**Keywords:** mycotoxin, cyclopiazonic acid, luminescence, lanthanides, mechanism of action, calcium-ATPase

## Abstract

Cycopiazonic acid (CPA) is a neurotoxin that acts through inhibition of the sarco(endo)plasmic reticulum Ca^2+^-ATPase (SERCA). CPA blocks the calcium access channel of the enzyme. The inhibition may involve the binding of CPA with a divalent cation such as Mg^2+^. The potential for CPA to act as a chelator also has implications for methods to detect this toxin. Certain of the lanthanide metals undergo a dramatic increase in luminescence upon coordination with small molecules that can transfer excitation energy to the metal. This report is the first to describe the coordination of CPA with lanthanide metals, resulting in a substantial enhancement of their luminescence. The luminescence expressed was dependent upon the type of lanthanide, its concentration, and the environment (solvent, water content, pH). Based upon the phenomenon, a competitive assay was also developed wherein terbium (Tb^3+^) and a series of metal cations competed for binding with CPA. With increasing cation concentration, the luminescence of the CPA/Tb^3+^ complex was inhibited. The chlorides of ten metals were tested. Inhibition was best with Cu^2+^, followed by Co^2+^, Al^3+^, Fe^3+^, Mn^2+^, Au^3+^, Mg^2+^, and Ca^2+^. Two cations in oxidation state one (Na^+^, K^+^) did not inhibit the interaction significantly. The interaction of CPA with lanthanides provides a novel recognition assay for this toxin. It also provides a novel way to probe the binding of CPA to metals, giving insights into CPA’s mechanism of action.

## 1. Introduction

**Figure 1:**
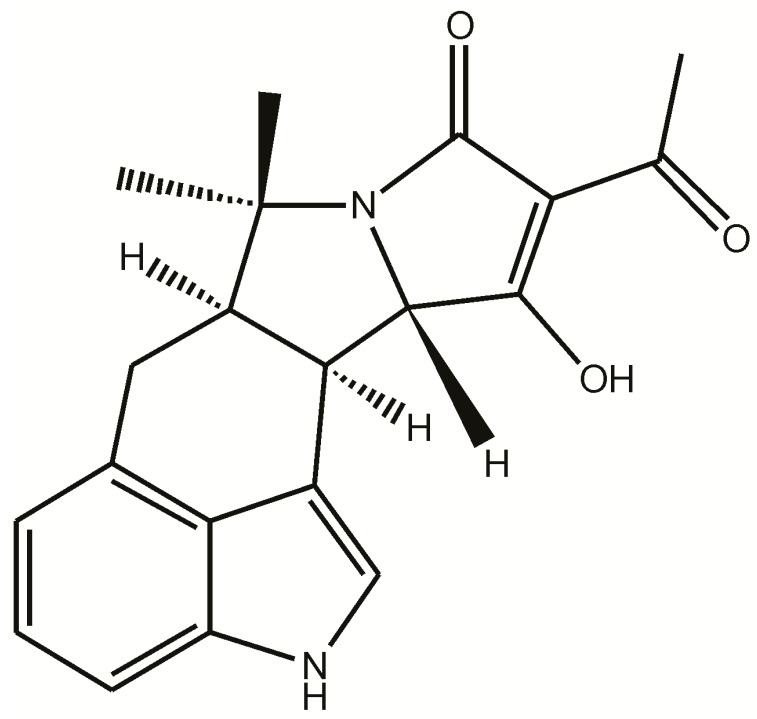
Structure of α-cyclopiazonic acid (CPA). Note the indole moiety capable of absorbing light at circa 280 nm, and the presence of the tetramic acid moiety that can coordinate with metal cations.

The mycotoxin α-cyclopiazonic acid (CPA) was first isolated from the fungus *Penicillium cyclopium* Westling fifty years ago by Holzapfel [1,2]. It is a low molecular weight compound classified as an indole tetramic acid (Figure 1). In the time since its discovery many of the fungi in the genera *Aspergillus* and *Penicillium* have been found to produce this neurotoxin [3]. CPA is frequently produced by some of the same fungi that produce the better known, and hepatocarcinogenic, aflatoxins [4,5,6]. In particular many strains of *Aspergillus flavus* can produce this toxin. This fungus can infest a variety of commodities and foods and, as a result, CPA has been found as a natural contaminant in many products including cheeses, figs, maize, rice, peanuts, millet, feeds [7,8,9,10,11,12] and chicken meat [13]. CPA may also have been a contributor to the original outbreak of “Turkey X” disease, which led to the discovery of the aflatoxins [14].

CPA can cause a variety of symptoms, which can vary by species. Symptoms may include weight loss, diarrhea, degeneration and necrosis of the muscles and viscera, leading to convulsions that can culminate in death [15]. The major target organs are the liver, kidneys, spleen, alimentary tract, lymphoid tissue, skeletal muscle, and the myocardium [15]. Male chickens that received CPA for 28 days showed dose-dependent decreases in the levels of Ca^2+^, Mg^2+^, and Fe^2+^ within their sera [16]. CPA is believed to act by disrupting calcium metabolism through inhibition of the sarcoplasmic reticulum Ca^2+^-ATPase (SERCA) [17,18,19]. SERCA expends ATP to move Ca^2+^ ions across the membrane. Crystallographic studies have suggested that CPA inhibits SERCA by blocking the calcium access channel, and that a divalent metal ion is required for binding [20,21]. A closely related Ca^2+^ pump, PfATP6, is also present in one of the protozoan parasites that cause malaria, *Plasmodium falciparum*. As such it is a potential anti-malarial target. A computational study of the binding of CPA within SERCA and PfATP6 revealed differences between the two that may be exploited to develop CPA-based derivatives more specific for the parasite [22].

Because of its toxicity and potential co-occurrence with aflatoxins, a variety of analytical techniques have been developed including chromatographic, mass spectrometric (MS), and immunochemical methods [2,3,23]. High performance liquid chromatography (HPLC) methods for CPA have generally taken advantage of the absorbance of the toxin in the ultraviolet (UV) range at circa 280 nm and often include an agent such as Zn(SO_4_) which complexes with CPA and improves chromatography [24]. CPA can also be detected through photoreaction to fluorescent products [25]. HPLC coupled to MS has become a very common analytical tool, and has been used to measure CPA in commodities [26,27,28]. Several immunoassays have also been developed to facilitate the rapid screening of commodities and foods [23,29,30]. However, the immunoassays for CPA are not commercially available, HPLC-MS requires expensive instrumentation and technical expertise, and the non-MS techniques require significant sample cleanup and pre-concentration of toxin before analysis. So, in spite of the significant advances made in CPA detection, further improvements in availability, cost, and ease of use remain desirable.

Early studies with CPA suggested the potential for it to occur naturally as a metal chelate complex [7]. More recently, crystallographic studies have suggested that CPA binds with a divalent cation, and this complex in turn inhibits SERCA [21]. Certain of the lanthanide metals, such as europium and terbium, can luminescence. This luminescence is generally very low, but can be greatly facilitated through interaction with molecules that can efficiently transfer energy to the lanthanide [31]. The phenomenon has led to the use of certain lanthanides as the basis of luminescent probes for molecular interactions. Lanthanides have been used as reagents for enhancing the luminescence of ochratoxin A (OTA) and citrinin in a post-column HPLC format [32] and in a DNA-aptamer-OTA biosensor [33].

A material that can transfer energy to a lanthanide, thereby enhancing its luminescence is known colloquially as an “antenna” because it receives energy and transmits it to the lanthanide. To function as an antenna, the molecule must absorb light and transmit it to the lanthanide in a fashion such that excitation can occur. This requires the coordination of the molecule with the lanthanide. The potential of CPA to form coordination complexes with divalent cations, the presence of an indole moiety with a UV absorption, and the proximity of the indole to the tetramic acid region of the molecule responsible for coordination with the cation, suggested that CPA might act as an “antenna” for enhancing lanthanide luminescence. The objective of the present research was to determine whether CPA might function in this manner and, if so, to determine the conditions under which the phenomenon could be optimized. Exploiting the phenomenon has two potential benefits. The first of these is the development of a new type of recognition assay for CPA that could be useful in biosensing applications. The second is that the phenomenon could be used to explore the binding of CPA to a variety of metal cations, thereby providing insights into the mechanism of action of this mycotoxin.

## 2. Results and Discussion

### 2.1. Binding of Lanthanides by CPA and Induction of Luminescence

There is a considerable body of literature describing how the luminescence of certain lanthanide metals such as europium and terbium can be enhanced. Generally this involves the transfer of energy from a closely associated molecule (known colloquially as an “antenna”) to the lanthanide. The process, known as sensitized emission, involves absorption of energy by the antenna, the inter system crossing of energy within the antenna, the transfer of energy to the lanthanide and, finally, the relaxation of the excited state of the lanthanide, resulting in luminescence [31]. In the best case scenario, the antenna itself undergoes little luminescence or phosphorescence, so that the excitation energy is efficiently transferred to the lanthanide. The lanthanides have large ionic radii, and have coordination numbers that are high: from 6 to 12 [34]. Coordination with water quenches luminescence from the lanthanide, so replacing lanthanide-water interactions can facilitate luminescence. These phenomena have led to the development of a large number of lanthanide-based chemosensors.

**Figure 2:**
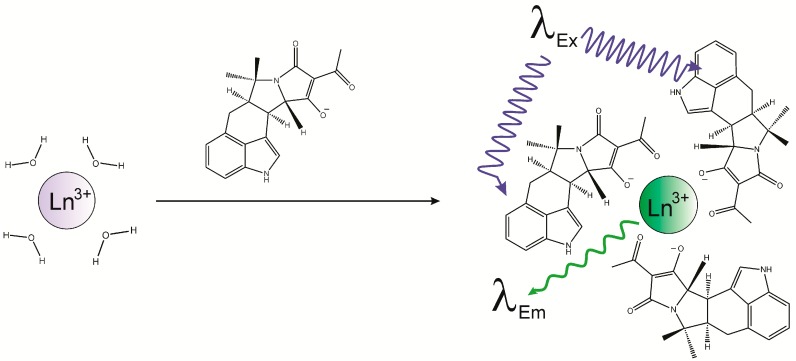
Formation of CPA-lanthanide complex and luminescence of the lanthanide. Excitation corresponds to an absorption band within CPA. Emission is from the lanthanide (Ln^3+^).

CPA has many of the attributes of a potential “antenna”. It has been reported that the tetramic acid portion of the molecule can chelate metals. The indole portion of the molecule has an ultraviolet (UV) absorption band which, in methanol, occurs at 284 nm and has an extinction coefficient of 20,417 [35]. Furthermore the indole is located in close proximity to the tetramic acid moiety, suggesting the distance required for energy transfer to the metal would be relatively low. The potential benefits for exploring CPA binding to lanthanides are two-fold: the development of novel reagents that can facilitate the detection of CPA, and the possibility of providing insights into the mechanism of action of this toxin. For these reasons, an attempt was made to determine if CPA could bind with, and enhance the luminescence of, europium (Eu^3+^) and terbium (Tb^3+^). A schematic of a potential complex that could form from such an interaction is depicted in Figure 2. Preliminary experiments indicated that the luminescence of Eu^3+^ and Tb^3+^ were significantly enhanced in the presence of CPA. Further experiments were conducted to determine the parameters that influence this interaction and, following optimization, a competitive assay was established to allow for the determination of the relative affinity of 10 metal cations towards CPA.

The schematic in Figure 2 depicts several aspects of the interaction of CPA with lanthanides. In the presence of CPA the coordination of the Ln^3+^ with H_2_O has been replaced with coordination to CPA. Also depicted are the absorption of light by CPA, and emission from the lanthanide. Although not depicted, in order for luminescence to occur there must also be intersystem transfer of energy from CPA to the lanthanide. While certain substituted indoles can be fluorescent themselves, CPA is not-fluorescent unless it undergoes photolysis [25]. Importantly, lanthanide ions have been reported to quench the fluorescence of the indole ring system. The latter is significant, because it has been suggested to occur through a mechanism involving electron transfer from the indole ring to the lanthanide [36]. Essentially the energy is transferred from the indole to the lanthanide, which is observed as quenching of the indole and excitation of the lanthanide. The existence of intersystem crossing suggests that the resulting emission from the lanthanide should be categorized as phosphorescence rather than fluorescence [34]. Because differentiating between the two requires measurement of the emission lifetimes, which is beyond the scope of this manuscript, the process herein is referred to as luminescence because the latter term is more general and includes both fluorescence and phosphorescence.

### 2.2. Importance of the Lanthanide

**Figure 3:**
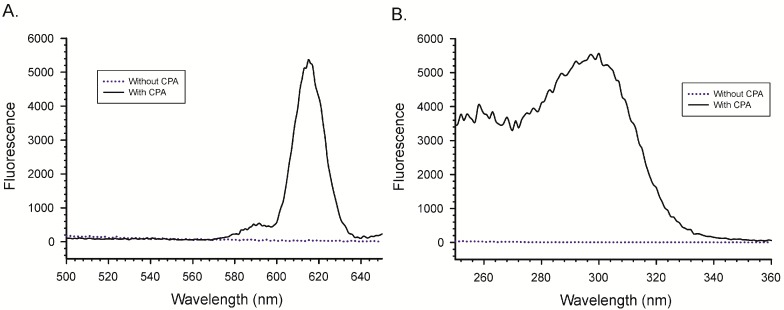
Effect of CPA on the luminescence of Eu^3+^. (**A**) Emission spectrum with excitation at 290 nm; (**B**) Excitation spectrum with emission at 615 nm. All spectra were collected in MeOH/H_2_O (9 + 1).

To determine which lanthanide to use, EuCl_3_ and TbCl_3_ were each reacted with CPA and then scanned for the optimal excitation and emission wavelengths for maximal luminescence. In order to determine which lanthanide might be most sensitive to the presence of CPA, the experiments were conducted at relatively low concentrations of reactants. The results for EuCl_3_ are depicted in Figure 3. Depicted are the excitation and emission spectra obtained with 2.5 µM Eu^3+^ and 25 µM CPA. It is clear from the figure that, in the absence of CPA, there was no detectable luminescence from the Eu^3+^. The emission maximum for the Eu^3+^/CPA complex was 615 nm, while the excitation maximum was at 290–300 nm.

**Figure 4:**
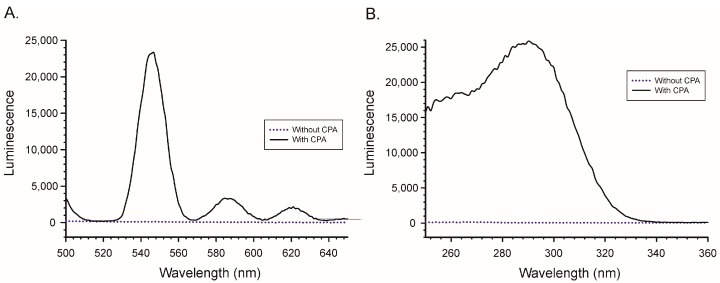
Effect of CPA on the luminescence of Tb^3+^. (**A**) Emission scan with excitation at 290 nm; (**B**) Excitation scan with emission at 545 nm. All scans collected in MeOH/H_2_O (9 + 1).

Similarly, the presence of CPA was needed in order to observe luminescence from Tb^3+^ (Figure 4). In this case much lower concentrations of lanthanide (0.25 µM) and CPA (1.5 µM) were required to see the effect. The emission maximum for the Tb^3+^/CPA complex was 545 nm, while the excitation maximum was 290 nm. While the concentrations of Tb^3+^ used in Figure 4 were much lower than the concentrations of Eu^3+^ used in Figure 3, the signals were roughly 4-fold greater. This combination of lower reagents and higher signal suggested that CPA was much better at enhancing the luminescence of Tb^3+^ than Eu^3+^. For this reason Tb^3+^ was selected for use in the remainder of the experiments.

### 2.3. Effects of Lanthanide Concentration

**Figure 5:**
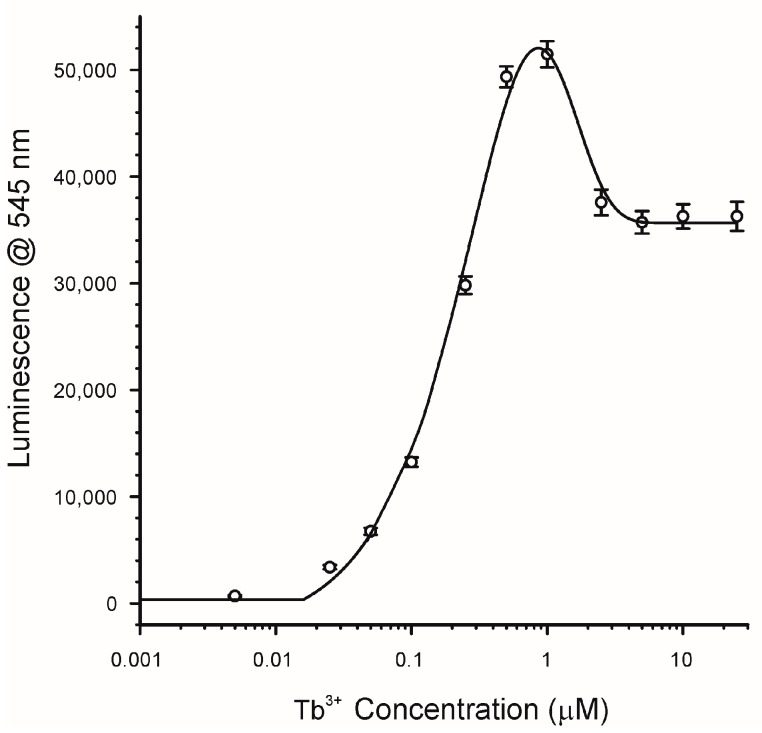
Dependence of luminescence upon the TbCl_3_ concentration. CPA was present at 1.5 uM (504 ng/mL). Excitation at 290 nm, emission at 545 nm. Points represent the average of triplicate plates with 8 wells per concentration (*n* = 24) ± 1 standard deviation.

Terbium and europium are well known to form coordination complexes with a wide range of coordination geometries. Coordination numbers typically range from 6 to 12 [31,34], which gives the potential to coordinate with multiple CPA, as well as water and solvent molecules. The schematic shown in Figure 2 represents only one of the many possible coordination complexes. Multiple coordination is a hallmark of many of the reported sensors that incorporate lanthanides [31,37]. To explore the potential for multiple coordination and to optimize the conditions under which luminescence of Tb^3+^ was enhanced by CPA, the effect of the Tb^3+^ concentration was examined. The results are depicted in Figure 5. In this experiment the concentration of CPA was kept constant (1.5 µM) and the concentration of TbCl_3_ was increased. Because the CPA was held constant, this figure also shows the effect of decreasing the molar ratio of CPA:Tb^3+^. As might be expected there was initially a rapid increase in response as the concentration of Tb^3+^ increased and the CPA:Tb^3+^ ratio decreased. This was the range over which the concentration of Tb^3+^ was limiting. However, at concentrations of circa 0.5 to 1 µM TbCl_3_, the responses peaked. This corresponds to a ratio of CPA:Tb^3+^ of 3:1 (at 0.5 µM) and 1.5:1 (at 1 µM). It is suggested therefore that the optimal coordination of CPA with Tb^3+^ occurs in the range of 1.5 to 3 CPA per Tb^3+^. This is the reason why Figure 2 is depicted as a complex of 3:1 CPA:Tb^3+^. Notably, as the concentration of Tb^3+^ was increased further the luminescence response actually decreased. The effect was seen at Tb^3+^ concentrations of 2.5 µM and above, corresponding to ratios of CPA:Tb^3+^ of less than 0.6. At higher concentrations of TbCl_3_ the Tb^3+^ is in excess and the luminescence reaches a plateau (Figure 5). The fact that this plateau occurs at ratios of CPA:Tb^3+^ of less than 0.6, suggests optimal response is achieved when multiple CPA are available to interact with each Tb^3+^.

### 2.4. Effects of Environment

As a general phenomenon, the emission from fluorophores can often be impacted dramatically by their environment, in particular the polarity of the surrounding solvent and the presence of water. Specifically, it is known that water quenches the emission from lanthanides. For this reason the interaction of CPA with Tb^3+^ was examined in a variety of solvents and solvent/water mixtures.

#### 2.4.1. Solvent Type

**Figure 6:**
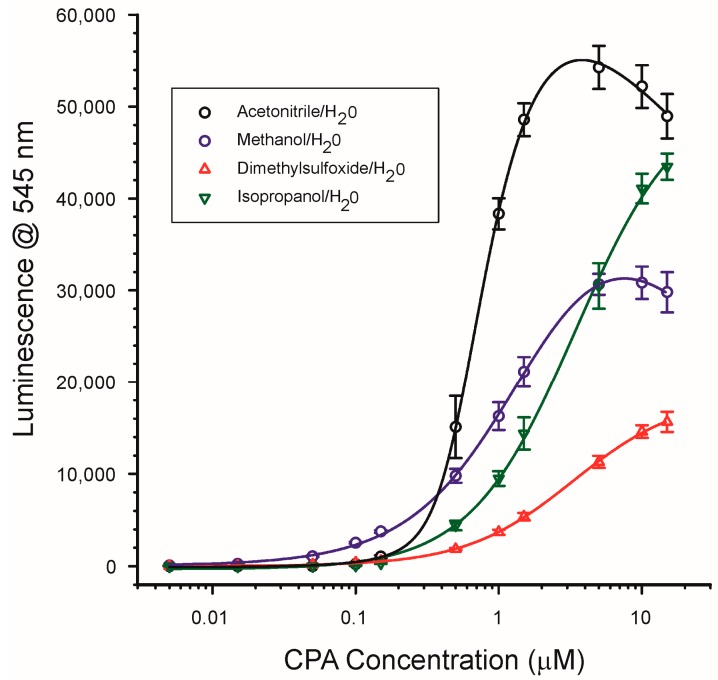
Effect of solvent type upon the luminescence of CPA/Tb^3+^. Solvents shown were all mixtures of 9 + 1 (*v*/*v*) with H_2_O. TbCl_3_ was present at 0.25 uM. Excitation at 290 nm, emission at 545 nm. Points represent the average of triplicate plates with 8 wells per concentration (*n* = 24) ± 1 standard deviation. One µM CPA is equivalent to 336 ng/mL.

The effects of solvent type on the CPA/Tb^3+^ interaction were investigated using four polar solvents, two of which were protic (methanol, isopropanol) and two of which were non-protic (DMSO, ACN). To ensure that metal chlorides would be soluble in the resulting solutions (Section 2.5), even at levels as high as 50 mM, the solvents were tested as 9 + 1 mixtures of solvent + water. For these experiments calibration curves of CPA were prepared in the indicated solvent mixtures with TbCl_3_ present at 0.25 µM and CPA present at concentrations ranging from 0.005 to 15 µM (1.68 to 5040 ng/mL). The solvent selected clearly had a significant influence upon the sensitivity of the assay and the shape of the calibration curve (Figure 6). The greatest overall signals were observed with ACN/H_2_O and IPOH/H_2_O, with DMSO/H_2_O the poorest. The different shapes of the calibration curves were of interest. The curves in ACN/H_2_O and MeOH/H_2_O showed a clear maximum signal at circa 3 to 8 µM CPA. Above these concentrations, the luminescence actually appeared to decrease. Unlike the case for the decrease in response observed with excess Tb^3+^ the reason for the decrease with excess CPA is unclear. The effect was not seen with the other two solvent mixtures: IPOH/H_2_O and DMSO/H_2_O which displayed calibration curves where the response did not appear to have reached a maximum even at the highest CPA concentration tested (15 µM).

The calibration curve in MeOH/H_2_O (9 + 1) was particularly interesting, because this solvent mixture gave the best responses for CPA at concentrations below 0.5 µM (Figure 6). At the lowest concentration tested (10 nM added, or 5 nM in the test solution) the signal to noise ratio was 5.9. This concentration, equal to 3.36 ng/mL added, suggested the system was very sensitive for detecting CPA. For comparison, a recent immunoassay for CPA demonstrated a limit of quantification of 0.71 nM (0.24 ng/mL) [23]. While not as sensitive as the immunoassay method, the CPA/Tb^3+^ system was very rapid and easy to perform, suggesting that further optimization was warranted. The ability to detect CPA binding at low concentrations was the reason that MeOH was selected as the base solvent for which to determine the effects of water and pH upon the assay.

#### 2.4.2. Water Content and pH

**Figure 7:**
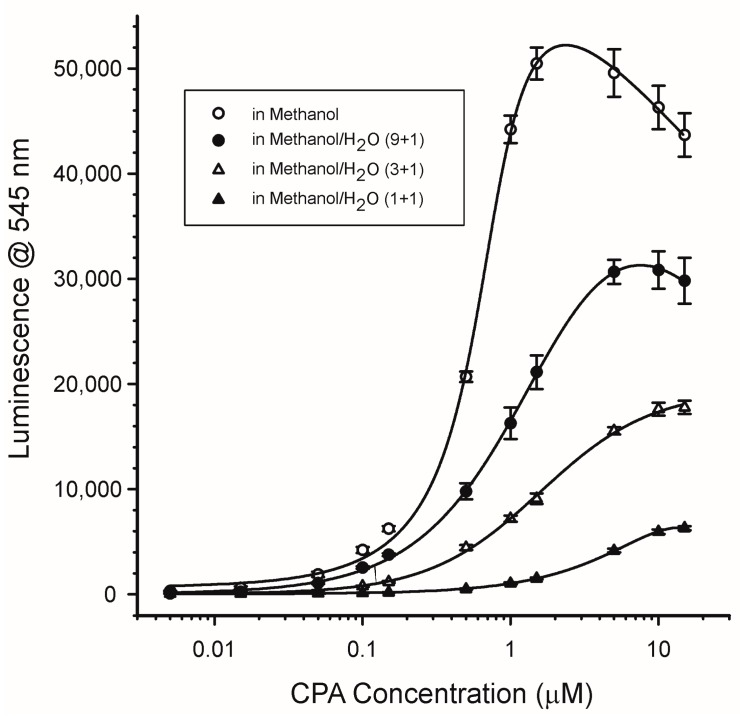
Effect of water on the luminescence of CPA/Tb^3+^. TbCl_3_ was present at 0.25 µM. Excitation at 290 nm, emission at 545 nm. Points represent the average of triplicate plates with 8 wells per concentration (*n* = 24) ± 1 standard deviation.

Because coordination with water is known to quench many fluorophores, the impact of water on the luminescence of the CPA/Tb^3+^ system was examined. In these experiments calibration curves of CPA were prepared in pure MeOH and MeOH to which water was added in the proportions of 9 + 1, 3 + 1, or 1 + 1 (*v*/*v*). The MeOH used to prepare these mixtures was reported to contain less than 0.05% water. The effect of water on the luminescence was dramatic (Figure 7). The greatest signals were seen with MeOH without added water. As the water content of the mixture increased, the maximal signals and sensitivity towards CPA declined. This is quite possibly due to the greater coordination of the Tb^3+^ with water at the expense of coordination with CPA. This suggested that, to maximize the luminescence of the CPA/Tb^3+^ system, minimizing the water concentration was important.

Ideally the effects of pH on the CPA/Tb^3+^ system would be evaluated over a wide pH range. To do so effectively would require using a variety of buffer species, for example acetate, phosphate, and carbonate. However, using multiple buffer types would introduce an additional variable: the counterion (acetate, phosphate, carbonate). Preliminary experiments with carbonate buffers at pH >8 demonstrated complete loss of the CPA/Tb^3+^ luminescence. In order to examine the effect of pH, a shorter pH range (3–7) was tested, with a single buffer species: 0.1 M acetate. Results indicated that the optimal pH was in the range of 3–4, with significant loss of response at pH 5 and above (Table 1).

Results of the pH experiment should be interpreted carefully. The pH values shown in Table 1 are those of the buffer measured before dilution 10-fold with MeOH. Dilution undoubtedly affected the actual pH of the test solution and would render inaccurate any pH measurements made of the resulting 90% MeOH solution. These results demonstrate that the luminescence observed with the CPA/Tb^3+^ system depends upon the complex interactions of multiple variables, including solvent type, water content, pH, and the identity and concentration of buffer species that may be present.

### 2.5. Competitive Luminescence Assay and Effects of Metal Cations

**Figure 8:**
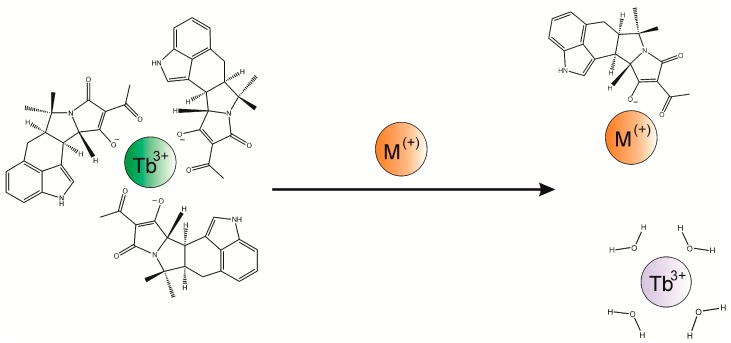
Competitive inhibition of the association of CPA and Tb^3+^ with metal cations. In sufficient excess the metal cations (shown as “M^+^”) can replace the Tb^3+^. The resulting decrease in emission results from the dissociation of the CPA from the Tb^3+^, which reduces absorption of excitation light by the Tb^3+^. Furthermore, coordination of Tb^3+^ with water results in quenching.

The ability to sensitively detect the binding of CPA to Tb^3+^ provided a tool for probing the interaction of other metal cations with CPA. Specifically, the formation of a CPA/Tb^3+^ complex provided the opportunity to explore how other metal cations, with various oxidation states, might disrupt the complex and impact luminescence. Rather than measuring the metal ions, the concept was to determine which metals interacted the best with CPA. The principle of this competitive assay is depicted in Figure 8.

**Figure 9:**
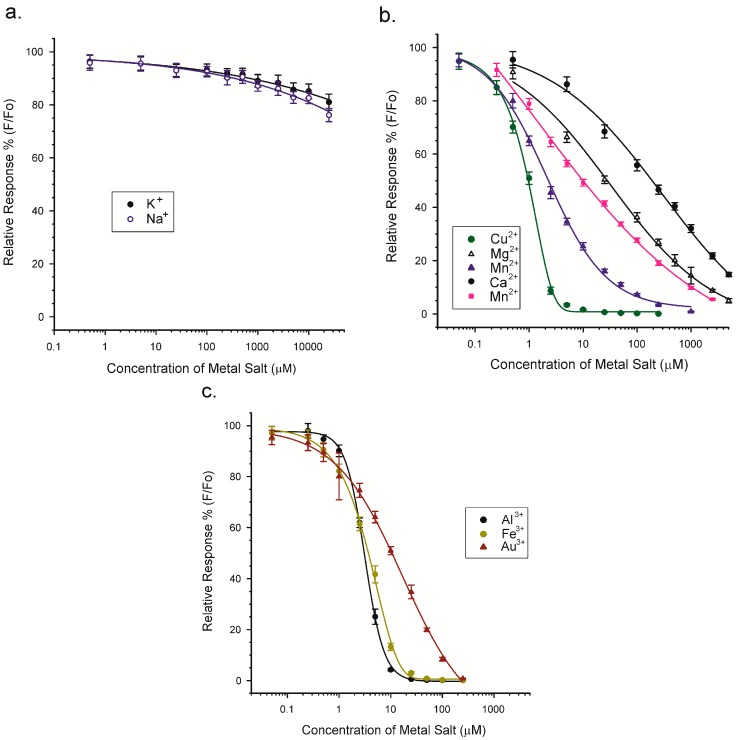
Competition between various metals and Tb^3+^ for CPA. (**a**) Metals with an oxidation state of one; (**b**) Metals with an oxidation state of two; (**c**) Metals with an oxidation state of three. Data expressed as the percentage of luminescence (F) relative to that seen in the absence of added metal salt (Fo).

In these experiments the concentrations of CPA and Tb^3+^ were fixed (3 and 0.5 µM, respectively) and the concentration of the “competitor” metal chloride was increased over the range of 0.05 to 25,000 µM. To ensure the solubility of some of the metal chlorides that were tested, all of the experiments were conducted in MeOH/H_2_O (9 + 1), even though the sensitivity towards CPA would have been greater in pure MeOH. The concentrations of CPA and Tb^3+^ that were used were selected in order to give a good signal (30,000 to 40,000) in MeOH/H_2_O. The ratio of CPA:Tb^3+^ was 6:1, which was higher than the optimal of approximately 3:1 shown in Figure 5. This puts the ratio used in the range where the Tb^3+^, rather than CPA, was the limiting reagent in the development of luminescence. Results for 10 metal chlorides are depicted in Figure 9.

Clearly the type and oxidation state of the metal had an impact on the ability to interact with CPA and disrupt the CPA/Tb^3+^ complex. The two metals in oxidation state one (K^+^, Na^+^) failed to disrupt the interaction of CPA with Tb^3+^, except at very high salt concentrations (>10 mM). Metal cations of oxidation state two or three were much more effective at disrupting the interaction. The concentrations of metal chloride that caused 50% inhibition of the CPA/Tb^3+^ luminescence (IC_50_) are provided in Table 2.

From these results it was apparent that Cu^2+^ was the best at disrupting the CPA/Tb^3+^ interaction. The concentration of Cu^2+^ at the IC_50_ was approximately 1 µM, in comparison to the 1.5 µM CPA and 0.25 µM Tb^3+^ which were present. This suggests a strong affinity of CPA for Cu^2+^. Using this metric the relative affinities of CPA for the 10 cations were: Cu^2+^, Co^2+^, Al^3+^, Fe^3+^, Mn^2+^, Au^3+^, Mg^2+^, and Ca^2+^, with very little affinity for Na^+^ and K^+^. There was no clear distinction between metals in oxidation states two and three. The reasons behind these relative affinities might be revealed through molecular modeling studies of the various metal/CPA coordination complexes.

The ability of CPA to bind and interact with cations is relevant to its mechanism of action as a neurotoxin. CPA is thought to inhibit SERCA through the formation a complex with a divalent cation within the Ca^2+^ access channel. The sequestration of Ca^2+^ by CPA has not been widely regarded as a factor in its toxicity. The experiments herein, whereby the various metals competed with Tb^3+^ for binding to CPA indicated that Ca^2+^ was a relatively poor competitor (Table 2). This supports the concept that chelation of Ca^2+^ is not a primary mode of action of CPA. However, high intracellular concentrations of Ca^2+^ or the absence of other competing divalent and trivalent cations might change that interpretation. A crystallographic study of the SERCA-CPA complex determined that a divalent metal ion was present with the CPA. In that case, MnCl_2_ was used in the crystallization buffer, and the complex found contained Mn^2+^ [21]. Intriguingly, in our study, Mn^2+^ was also a much better inhibitor of the CPA/Tb^3+^ complex than Ca^2+^. The results from our study also raise the interesting possibility that, by binding with CPA, other divalent or trivalent cations might influence its ability to bind SERCA and therefore potentially its toxicity. Although the latter is highly speculative, the possibility does suggest that future studies of the toxicity of CPA should consider the potential impact of the metal ions that are present.

## 3. Conclusions

The ability of CPA to interact with europium and terbium, resulting in the formation of luminescent complexes, was demonstrated. Of the two lanthanides, the enhancement of luminescence was greater with terbium. The environment surrounding the complex was important, with the selection of solvent, water content, and pH having a significant impact on the observed luminescence. In a 9 + 1 mixture of MeOH/H_2_O, as little as 10 nM (3.4 ng/mL) of CPA was detected. In order to study the relative affinity of CPA for various metal cations, a competitive assay was designed, with results suggesting that the oxidation state, while important, was not the only factor important for interaction with CPA. The Tb^3+^/CPA system should be a useful tool for further studies of the interactions of CPA with cations.

## 4. Materials and Methods

### 4.1. Materials

Except where noted otherwise, deionized water (Nanopure II, Thermo Scientific, Waltham, MA, USA) was used in the preparation of all reagents. The CPA used as analytical standard was produced by MP Biomedicals, LLC (Solon, OH, USA). CPA stock solution was prepared at a nominal concentration of 2 mg/mL by dissolving solid toxin in HPLC grade acetonitrile. The actual concentration was determined by obtaining the ultraviolet (UV) spectrum of 1:200 dilutions of the stock in methanol (MeOH) (model DU640 spectrophotometer, Beckman Coulter, Brea, CA, USA) and using the extinction coefficient of 20,417 at 284 nm [1,35]. Terbium(III) chloride (TbCl_3_) hexahydrate, Europium(III) chloride (EuCl_3_) hexahydrate, Magnesium chloride (MgCl_2_) hexahydrate, Cobalt (II) chloride (CoCl_2_) hexahydrate, Copper(II) chloride (CuCl_2_) dehydrate, Ferric chloride (FeCl_3_) hexahydrate, Sodium chloride (NaCl), and Potassium chloride (KCl) were all purchased from Fisher Chemicals (Fair Lawn, NJ, USA). Gold (III) chloride (HAuCl_4_) hydrate, and Aluminum chloride (AlCl_3_) hexahydrate were purchased from Aldrich Chemical Company (Milwaukee, WI, USA). Anhydrous Calcium chloride (CaCl_2_) was purchased from J.T. Baker Chemical Company (Phillipsburg, NJ, USA). Acetonitrile (ACN), dimethylsulfoxide (DMSO), and isopropanol (IPOH) were HPLC grade and were purchased from Fisher. All other chemicals were reagent grade or better and purchased from major suppliers.

### 4.2. Excitation and Emission Spectra

Excitation and emission spectra of the CPA/lanthanide complexes were determined using a Neo microplate reader (BioTek, Winooski, VT, USA). All spectra were collected at 25 °C in MeOH/H_2_O (9 + 1) using normal (not time resolved) luminescence. Test volumes were 300 µL. Experiments were conducted in black microtiter plates (Corning, Inc., Kennebunk, ME, USA). General settings for the instrument included a gain setting of 150, scanning in 1 nm increments, with 50 measurements per increment. Lamp energy was set to “low”, and distance of the optics above the microplate was set to 4.5 mm. The read speed was “normal”. Emission scans were collected over the range of 500 to 650 nm at an excitation of 290 nm. Excitation scans were collected over the range of 250 nm to 360 nm with monitoring emission at either 545 nm (Tb^3+^) or 615 nm (Eu^3+^). To demonstrate the enhancement, excitation and emission spectra were collected with and without CPA. The concentrations that were used were determined empirically and were selected to use as little of the reagents as possible while still yielding spectra with significant signal (5000 counts per second or higher) to allow for direct comparisons of sensitivity. For Eu^3+^ this was with EuCl_3_ at 2.5 µM, with and without CPA at 25 µM. For Tb^3+^ this was with TbCl_3_ at 0.25 µM with and without CPA at 1.5 µM.

### 4.3. Effects of Lanthanide Concentration

A benefit of using lanthanide conjugates is their long luminescence lifetime, which permits their measurement using time resolved fluorescence (TRF). Aside from measurement of the excitation and emission maxima (Section 4.2), all experiments were conducted with TRF. For experiments on the effects of lanthanide concentration, 0.15 mL of CPA (at 3 µM in MeOH/H_2_0 9 + 1) and 0.15 mL of TbCl_3_ at 0.01 to 50 µM (also in MeOH/H_2_0 9 + 1) were mixed in the wells of black microtiter plates. The resulting solutions, 0.30 mL, contained 1.5 µM CPA and 0.005 to 25 µM TbCl_3_. Each experiment was conducted in triplicate: three separate microplates (with separately prepared standards) having 8 replicate wells at each concentration (*n* = 24). As with the spectral scanning, a Neo microplate reader was used. Wavelengths were selected by monochrometer. Unless noted otherwise, the excitation wavelength was 290 nm, and the emission wavelengths were either 545 nm (Tb^3+^) or 615 nm (Eu^3+^). Gain settings were 150, and the lamp was set to “low” energy. The top optics of the instrument were set to a height of 4.5 mm. TRF was monitored with a 100 µs delay after the lamp flash, and was collected for 500 µs.

### 4.4. Environmental Effects

#### 4.4.1. Effects of Solvent Type

The luminescence of the Tb^3+^/CPA complex was examined in different solvents, including ACN, MeOH, DMSO, and IPOH. For these experiments, the CPA and the TbCl_3_ were each dissolved in a mixture of 9 parts solvent and one part deionized water. CPA standards over the range of 0.01 to 30 µM were mixed with equal volumes of TbCl_3_ prepared at 0.5 µM in the same solvent/water mixture. Therefore the concentrations in the test mixtures were 0.25 µM TbCl_3_ and 0.005 to 15 µM CPA in 9 + 1 (solvent + H_2_O). Data were collected from triplicate microplates, as described in Section 4.3.

#### 4.4.2. Effects of Water Content and pH

The luminescence of the Tb^3+^/CPA complex was examined in pure methanol and in methanolic solutions containing various proportions of water. For these experiments, the CPA and the TbCl_3_ were each dissolved in a mixture of either MeOH, MeOH/H_2_O (9 + 1), MeOH/H_2_O (3 + 1), or MeOH/H_2_O (1 + 1). CPA standards over the range of 0.01 to 30 µM were prepared in a given MeOH/H_2_0 solution and were mixed with equal volumes of TbCl_3_ prepared at 0.5 µM in a solution with the same proportion of MeOH/H_2_O. Therefore final concentrations in the mixtures were 0.25 µM TbCl_3_ and 0.005 to 15 µM CPA in either: MeOH, MeOH/H_2_O (9 + 1), MeOH/H_2_O (3 + 1), or MeOH/H_2_O (1 + 1). To determine the effects of pH, only the ratio of 9 + 1 MeOH/buffer was used. The buffers were 0.1 M acetic acid, adjusted to cover the pH range of 3 to 7 with sodium hydroxide. MeOH/buffer at an indicated pH was used to prepare solutions containing a mixture of 0.5 µM TbCl_3_ and 3 µM CPA. Data were collected from triplicate microplates, as described in Section 4.3.

### 4.5. Competitive Luminescence Assay

To determine the relative affinity of CPA for various metal cations, the chlorides of 10 metals were added to a mixture of CPA and TbCl_3_. The inhibition of luminescence of the CPA/Tb^3+^ was determined over a wide range of concentrations of the added metal chloride. The range of concentrations tested were adjusted depending upon the ability of the corresponding metal to inhibit the CPA/Tb^3+^ interaction. In these experiments a solution was prepared containing 0.5 µM TbCl_3_ and 3.0 µM CPA in MeOH/H_2_0 (9 + 1). In wells of a black microtiter plate, 0.15 mL of this solution was mixed with 0.15 mL of the various metal chlorides, also prepared in MeOH/H_2_0 (9 + 1). The metal chlorides were prepared at concentrations ranging from 0.1 to 50,000 µM. The concentrations present in the test mixtures were therefore 0.25 µM TbCl_3_ and 1.5 µM CPA, with metal chloride over the range of 0.05 to 25,000 µM. Data were collected from triplicate microplates, as described in Section 4.3. To facilitate comparisons among ions the data were normalized for the luminescence of the system in the absence of added competitor, that is, F/Fo where F was the observed luminescence and Fo was the luminescence of the CPA/TbCl_3_ mixture in the absence of competitor.

### 4.6. Data Analysis and Curve Fitting

Data from individual microplates (8 replicate wells per concentration) were averaged and were analyzed with TableCurve 2D [38]. For determining the effects of lanthanide concentration and environmental conditions (solvent type, water content), curves were selected that fit the data reasonably well (*r^2^* > 0.99), but which were not necessarily the best fitting curves. Curve fitting software can generate extremely well fitting curves which often simply connect the data point-to-point. Because of this an attempt was made to select fitting equations that were fairly general and not specific to individual curves. The variety of curve shapes dictated that several different fitting equations be used, and three were selected for general use. For fitting the data of the effects of TbCl_3_ concentration (Section 4.3), where there is a distinct peak followed by a plateau, a six paramenter Equilibrium Peak Function was used (TableCurve equation # 8071). For determining the effects of solvent type (Section 4.4.1), the acetonitrile/H_2_O and MeOH/H_2_O data were fit with a six parameter Pearson IV equation (TableCurve #8187), while the DMSO/H_2_O and IPOH/H_2_O data were fit with a logistic dose-response equation (TableCurve #8013). For determining the effects of water content (Section 4.4.2) The data obtained in pure MeOH and MeOH/H_2_O (9 + 1) were also fit with the Pearson IV equation (#8187), while the data from MeOH/H_2_O at proportions of (3 + 1) and (1 + 1) were fit with the logistic dose-response equation (#8013). For the competitive assays, Section 4.5, the equations used to fit the data are summarized in Table 1, which include both equations 8187, 8013, and an additional equation, #8007, which is a 4 parameter logistic peak function. The equations and the parameters involved are described in detail in the TableCurve 2D User’s Manual [38].

## Figures and Tables

**Table 1:** Effect of pH on the CPA/Tb^3+^ system.

pH	Luminescence ^a^
3.0	42,250 ± 1590
4.0	44,170 ± 2540
5.0	23,270 ± 1850
6.0	12,200 ± 940
7.0	1560 ± 140

^a^ Average of 16 measurements on triplicate plates (*n* = 48) ± 1 standard deviation. CPA present at 3 µM and Tb^3+^ present at 0.5 µM. The pH was measured before dilution 10-fold with MeOH.

**Table 2:** Competitive inhibition of the binding of CPA to Tb^3+^ with metal cations.

Cation	IC_50_ (µM) ^a^	Molar Ratio of Cation: CPA at the IC_50_^b^	Equation ^c^
Cu^2+^	0.98 ± 0.06	0.65	8187
Co^2+^	2.29 ± 0.14	1.52	8013
Al^3+^	3.08 ± 0.13	2.05	8013
Fe^3+^	3.71 ± 0.33	2.47	8007
Mn^2+^	9.98 ± 0.45	6.65	8013
Au^3+^	10.7 ± 0.07	7.1	8013
Mg^2+^	28.3 ± 1.0	18.8	8013
Ca^2+^	191 ± 19	127	8013
Na^+^	>25,000	>16,667	NA ^d^
K^+^	>25,000	>16,667	NA

^a^ Average of triplicate plates ± 1 standard deviation. ^b^ CPA present at a concentration of 1.5 µM and Tb^3+^ present at 0.25 µM. ^c^ Equation used to fit the calibration curve, using TableCurve. See text for description of the curve types. ^d^ NA: not applicable. Could not be determined because concentrations required were too high.

## References

[1] Holzapfel C.W. (1968). The isolation and structure of cyclopiazonic acid, a toxic metabolite of *Penicillium cyclopium* Westling. Tetrahedron.

[2] Ostry V., Toman J., Grosse Y., Malir F. (2018). Cyclopiazonic acid: 50th anniversary of its discovery. World Mycotoxin J..

[3] Dorner J.W., DeVries J., Trucksess M.W., Jackson L.S. (2002). Recent advances in analytical methodology for cyclopiazonic acid. Mycotoxins in Food Safety.

[4] Trucksess M.W., Mislivec P.P., Young K., Bruce V.R., Page S.W. (1987). Cyclopiazonic acid production by cultures of *Aspergillus* and *Penicillium* species isolated from dried beans, corn meal, macaroni, and pecans. J. AOAC Int..

[5] Goto T., Wicklow D.T., Ito Y. (1996). Aflatoxin and cyclopiazonic acid production by a sclerotium-producing *Aspergillus tamarii* strain. Appl. Environ. Microbiol..

[6] Horn B.W., Dorner J.W. (1999). Regional differences in production of aflatoxin B_1_ and cyclopiazonic acid by soil isolates of *Aspergillus flavus* along a transect within the United States. Appl. Environ. Microbiol..

[7] Gallagher R.T., Richard J.L., Stahr H.M., Cole R.J. (1978). Cyclopiazonic acid production by aflatoxigenic and non-aflatoxigenic strains of *Aspergillus flavus*. Mycopathologia.

[8] Lansden J.A., Davidson J.I. (1983). Occurrence of cyclopiazonic acid in peanuts. Appl. Environ. Microbiol..

[9] Urano T., Trucksess M.W., Matuskik J., Dorner J.W. (1992). Liquid chromatographic determination of cyclopiazonic acid in corn and peanuts. J. AOAC Int..

[10] Burdock G.A., Flamm W.G. (2000). Review article: Safety assessment of the mycotoxin cyclopiazonic acid. Int. J. Toxicol..

[11] Hayashi Y., Yoshizawa T. (2005). Analysis of cyclopiazonic acid in corn and rice by a newly developed method. Food Chem..

[12] Heperkan D., Somuncuoglu S., Karbancioglu-Güler F., Mecik N. (2012). Natural contamination of cyclopiazonic acid in dried figs and co-occurrence of aflatoxin. Food Control.

[13] Miller C.D., Richard J.L., Osweiler G.D. (2011). Cyclopiazonic acid toxicosis in young turkeys: Clinical, physiological, and serological observations. Toxin Rev..

[14] Cole R.J. (1986). Etiology of turkey X disease in retrospect: A case for the involvement of cyclopiazonic acid. Mycotoxin Res..

[15] Voss K.A., Llewellyn G.C., O’Rear C.E. (1990). In vivo and in vitro toxicity of cyclopiazonic acid (CPA). Mycotoxins, Biotoxins, Wood Decay, Air Quality, Cultural Properties, General Biodeterioration, and Degradation.

[16] Akbari P., Malekinejad H., Rahmani F., Rezabakhsh A., Fink-Gremmels J. (2012). Cyclopiazonic acid attenuates the divalent cations and augments the mRNA level of iNOS in the liver and kidneys of chickens. World Mycotoxin J..

[17] Goeger D.E., Riley R.T., Dorner J.W., Cole R.J. (1988). Cyclopiazonic acid inhibition of the Ca^2+^-transport ATPase in rat skeletal muscle sarcoplasmic reticulum vesicles. Biochem. Pharmacol..

[18] Takahashi S., Kato Y., Adachi M., Agata N., Tanaka H., Shigenobu K. (1995). Effects of cyclopiazonic acid on rat myocardium: Inhibition of calcium uptake into sarcoplasmic reticulum. J. Pharmacol. Exp. Ther..

[19] Riley R.T., Goerger D.E., Norred W.P., Eklund M., Richard J.L., Mise K. (1995). Disruption of calcium homeostasis: The cellular mechanism of cyclopiazonic acid toxicity in laboratory animals. Molecular Approaches to Food Safety Issues Involving Toxic Microorganisms.

[20] Moncoq K., Trieber C.A., Young H.S. (2007). The molecular basis for cyclopiazonic acid inhibition of the sarcoplasmic reticulum calcium pump. J. Biol. Chem..

[21] Laursen M., Bublitz M., Moncoq K., Olesen C., Møller J.V., Young H.S., Nissen P., Morth J.P. (2009). Cyclopiazonic acid is complexed to a divalent metal ion when bound to the sarcoplasmic reticulum Ca^2+^-ATPase. J. Biol. Chem..

[22] Di Marino D., Ilda D.A., Andrea C., Allegra V., Anna T. (2015). Characterization of the differences in the cyclopiazonic acid binding mode to mammalian and *P. falciparum* Ca^2+^ pumps: A computational study. Prot. Struct. Funct. Bioinform..

[23] Maragos C.M., Sieve K.K., Bobell J. (2017). Detection of cyclopiazonic acid (CPA) in maize by immunoassay. Mycotoxin Res..

[24] Da Motta S., Valente Soares L.M. (2000). Simultaneous determination of tenuazonic and cyclopiazonic acids in tomato products. Food Chem..

[25] Maragos C.M. (2009). Photolysis of cyclopiazonic acid to fluorescent products. World Mycotoxin J..

[26] Moldes-Anaya A.S., Asp T.N., Eriksen G.S., Skaar I., Rundberget T. (2009). Determination of cyclopiazonic acid in food and feeds by liquid chromatography-tandem mass spectrometry. J. Chromatogr. A.

[27] Diaz G.J., Thompson W., Martos P.A. (2010). Stability of cyclopiazonic acid in solution. World Mycotoxin J..

[28] Ansari P., Haeubl G. (2016). Determination of cyclopiazonic acid in white mould cheese by liquid chromatography-tandem mass spectrometry (HPLC-MS/MS) using a novel internal standard. Food Chem..

[29] Hahnau S., Weiler E.W. (1993). Monoclonal antibodies for the enzyme immunoassay of the mycotoxin cyclopiazonic acid. J. Agric. Food Chem..

[30] Yu W., Chu F.S. (1998). Improved direct competitive enzyme-linked immunosorbent assay for cyclopiazonic acid in corn, peanuts, and mixed feed. J. Agric. Food Chem..

[31] Aulsebrook M.L., Graham B., Grace M.R., Tuck K.L. (2017). Lanthanide complexes for luminescence-based sensing of low molecular weight analytes. Coord. Chem. Rev..

[32] Vazquez B.I., Fente C., Franco C., Cepeda A., Prognon P., Mahuzier G. (1996). Simultaneous high-performance liquid chromatographic determination of ochratoxin A and citrinin in cheese by time-resolved luminescence using terbium. J. Chromatogr. A.

[33] De Girolamo A., Le L., Penner G., Schena R., Visconti A. (2012). Analytical performances of a DNA-ligand system using time-resolved fluorescence for the determination of ochratoxin A in wheat. Anal. Bioanal. Chem..

[34] Heine J., Műller-Buschbaum K. (2013). Engineering metal-based luminescence in coordination polymers and metal-organic frameworks. Chem. Soc. Rev..

[35] Nesheim S., Stack M.E., Trucksess M.W., Pohland A.E. (2001). Preparation of mycotoxin standards. Mycotoxin Protocols.

[36] Ricci R.W., Kilichowski K.B. (1974). Fluorescence quenching of the indole ring system by lanthanide ions. J. Phys. Chem..

[37] Aletti A.B., Gillen D.M., Gunnlaugsson T. (2018). Luminescent/colorimetric probes and (chemo-) sensors for detecting anions based on transition and lanthanide ion receptor/binding complexes. Coord. Chem. Rev..

[38] Systat Software, Inc. (2002). TableCurve 2D User’s Manual Version 5.01 for Windows.

